# Hydrolysis Activity of Virgin Coconut Oil Using Lipase from Different Sources

**DOI:** 10.1155/2018/9120942

**Published:** 2018-01-14

**Authors:** T. A. V. Nguyen, Truong D. Le, Hoa N. Phan, Lam B. Tran

**Affiliations:** ^1^Industrial University of Ho Chi Minh City, Ho Chi Minh City, Vietnam; ^2^Bach Khoa University, Ho Chi Minh City, Vietnam

## Abstract

Two types of lipase,* Candida rugosa* lipase (CRL) and porcine pancreas lipase (PPL), were used to hydrolyze virgin coconut oil (VCO). The hydrolysis process was carried out under four parameters, VCO to buffer ratio, lipase concentration, pH, and temperature, which have a significant effect on hydrolysis of lipase. CRL obtained the best hydrolysis condition at 1 : 5 of VCO to buffer ratio, 1.5% of CRL concentration, pH 7, and temperature of 40°C. Meanwhile, PPL gave different results at 1 : 4 of VCO to buffer ratio, 2% of lipase concentration, pH 7.5, and 40°C. The highest hydrolysis degree of CRL and PPL was obtained after 16 hours and 26 hours, reaching 79.64% and 27.94%, respectively. Besides, the hydrolysis process was controlled at different time course (every half an hour) at the first 4 hours of reaction to compare the initial hydrolysis degree of these two lipase types. FFAs from hydrolyzed products were isolated and determined the percentage of each fatty acid which contributes to the FFAs mixture. As a result, medium chain fatty acids (MCFAs) made up the main contribution in composition of FFAs and lauric acid (C12) was the largest segment (47.23% for CRL and 44.23% for PPL).

## 1. Introduction

Virgin coconut oil (VCO) contains a great deal of MCFAs especially lauric acid (nearly 50%). There is a variety of benefits from MCFAs in VCO; for example, the MCFAs are digested easily and converted into energy in the liver directly. This makes MCFAs do not take part in the synthesis of cholesterol or deposit fat in body [1]. Moreover, Valente et al. (2017) showed that MCFAs in VCO can control overweight condition for women [2]. On the other hand, antibacterial ability of MCFAs is one of the remarkable abilities which is concerned by many authors. For example, Parfene et al. (2013) showed that MCFAs in crude coconut oil could inhibit* Yarrowia lipolytica* [3]. The experiments of Shilling et al. (2013) demonstrated the antimicrobial activity of VCO and MCFAs against* Clostridium difficile* [4]. Therefore, extracting FFAs from VCO is necessary to apply in further research because of their potential abilities.

There are many types of methods to release FFAs out of triglyceride structure such as acidic hydrolysis, alkaline hydrolysis, or enzymatic hydrolysis. Among of these methods, using enzymatic hydrolysis is a better way to protect the hydrolyzed product as well as keeping some properties of the product as desired. The enzymatic reaction is conducted under milder condition of temperature and pH when compared to acidic or alkaline hydrolysis. Thus, it can avoid some dangers from undesirable side-reactions or oxidization of products, having no effect on color of products [5]. Moreover, enzymatic hydrolysis requires no cofactor or any chemical additives as well as the reusable ability of enzyme [6]. The aim of this study was to determine the best hydrolysis conditions for two types of lipase from different sources:* Candida rugosa* lipase (CRL) and porcine pancreas lipase (PPL). Simultaneously, the hydrolysis degree was controlled every half an hour of the first 4 hours of hydrolysis reaction to compare the specific hydrolysis activity of these two types of lipase.

## 2. Materials and Methods

### 2.1. Materials

VCO was supported by Luong Quoi Coconut Co., Ltd (Ben Tre Province, Vietnam). CRL (Type VII, ≥700 unit/mg solid) and PPL (Type II, 100–500 units/mg protein) were from Sigma-Aldrich Co. (Canada). The primary chemicals in this research were potassium hydroxide, ethanol, isooctane, n-hexane, and the others which were purchased from Merck (Germany) with purification more than 95%.

Devices used for hydrolysis process were high speed homogenizer (IKA T 25 digital ULTRA-TURRAX, Germany), overhead stirrer (OS20, USA), and orbital shaker incubator (LM-2575RD) from Yihder Technology Co. (Taiwan). Evaporator (IKA RV digital V) from Germany was used to isolate FFAs, as well as GC-FID Shimadzu 2010 Plus for determination of FFAs composition.

### 2.2. Hydrolyzed VCO

Firstly, the mixture contained VCO and suitable buffer for different lipase types. The hydrolysis reaction of VCO by CRL used phosphate buffer while using borate buffer for PPL. Isooctane played a role in dissolving VCO with 1 : 1 of VCO to solvent ratio [7]. The emulsification process to create the substrates for lipase was conducted by a stirrer at speed of 10000 rpm in 15 minutes before adding the appropriate amount of lipase powder. Next, the mixture was stirred in 5 minutes to dissolve completely the lipase powder. The hydrolysis reaction was incubated in orbital shaker incubator device. The reaction was stopped and the hydrolysis degree (HD) was calculated after 2 hours of reaction as the following formula [8]:

(1) $$\begin{array}{c} HD=\frac{V_{KOH}*M_{KOH}*M_{FFAs}}{10*m}\left(\;\%\;\right), \end{array}$$

where *V*_KOH_ is the volume of titrated potassium hydroxide (KOH) (mL); *M*_KOH_ is the molarity of KOH solution (mol/L); *M*_FFAs_ is the average molecular weight of free fatty acids; *m* is mass of sample (g).

All the experiments were carried out in triplicate and data outcome was statistically analyzed by using R software.

### 2.3. FFAs Extraction

First of all, the released FFAs in the hydrolyzed mixture were neutralized to form the salt of FFAs by adding the excess amount of KOH. The isolating process of FFAs was conducted in separatory funnel using n-hexane as solvent to extract FFAs and residual glycerides. The upper phase of separatory funnel containing residual glycerides and n-hexane was taken out of the mixture and the FFAs in the lower phase were returned into the free form by adding the excess amount of 4 N HCl solution (pH value lower than 2). Then FFAs were extracted continuously by using n-hexane and purified by using rotary evaporator to remove n-hexane in the mixture [9].

### 2.4. Gas Chromatograph-Flame Ionization Detection Analysis

Gas chromatograph-flame ionization detection (GC-FID) was used in this study to determine the composition of FFAs. FFAs were converted into fatty acid methyl ester (FAME) before injecting 2 *μ*l of sample into the column by split injection mode with the split ratio 1 : 25. The column* DB-FFAP* (0.25 mm internal diameter, 30 m length, and 0.25 *μ*m film thickness) was used at pressure of 12 psi. Carrier gas used for this process was helium; FID and injector temperature were both at 250°C over the process. The initial temperature of the column was 100°C; temperature program was set up to 230°C at 7°C/min and maintained steady until the end of analysis process [10].

## 3. Results and Discussion

### 3.1. Evaluation of VCO to Buffer Ratio

Because catalytic activity of lipase is conducted on emulsion of oil and water, increasing the amount of buffer will have more links between oil and water which were formed as well as having more substrates for lipase to catalyze hydrolysis reaction, leading to the rise of hydrolysis degree. However, the excess amount of buffer will decline the catalytic ability of lipase significantly because there is the competition of substrate (binding of water to lipase prevents lipase from linking to substrates). As a result, the hydrolysis degree is decreased [7].

**Figure 1:**
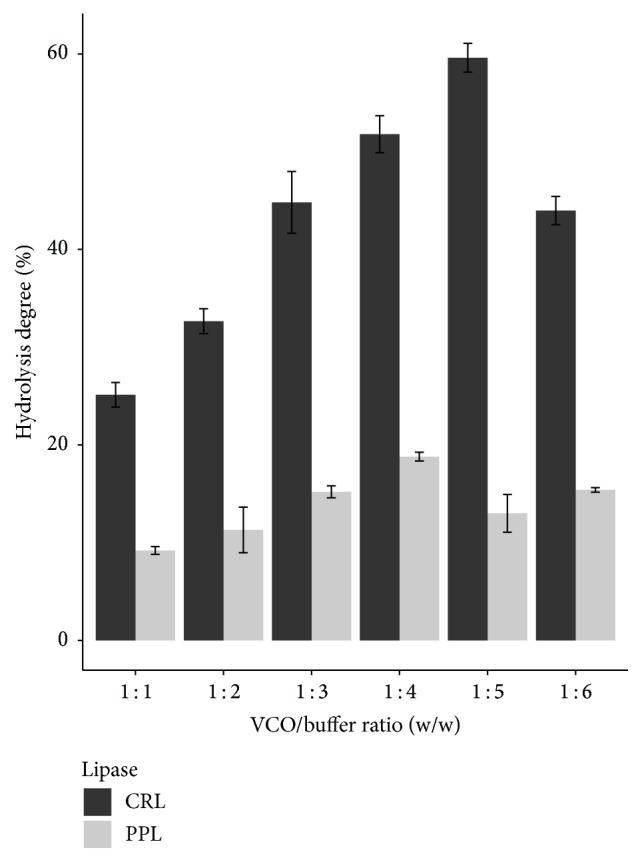
Effect of VCO to buffer ratio on hydrolysis degree of two lipase types.

According to Figure 1, each lipase had different VCO to buffer ratio. The highest hydrolysis degree was obtained at 1 : 4 VCO to buffer ratio for PPL, whereas CRL needed the VCO to buffer ratio up to 1 : 5.

In general, oil to buffer ratio was not equivalent to other studies due to the substrate specificity of lipase and different hydrolysis methods. The solubility of lipase and linking to ester bond of triglycerides are different because of different kinds of oil and the methods used for hydrolysis reaction, leading to a variety of adding the amount of buffer. For example, the study of Sharma et al. (2013), using CRL to hydrolyze cod liver oil was conducted at 1 : 4 of oil to buffer ratio and isooctane was used as solvent to dissolve cod liver oil [7]. Meanwhile, Freitas et al. (2007) used CRL and PPL to hydrolyze soybean oil with 1 : 4 of oil to buffer ratio with the support of 2.5% of Arabic gum instead of using isooctane in the mixture [11].

### 3.2. Evaluation of Lipase Concentration

Adding more lipase concentration leads to the increase in hydrolysis degree. However, if lipase is saturated with the substrate, the higher lipase concentration will not make the significant rise in hydrolysis degree [12].

**Figure 2:**
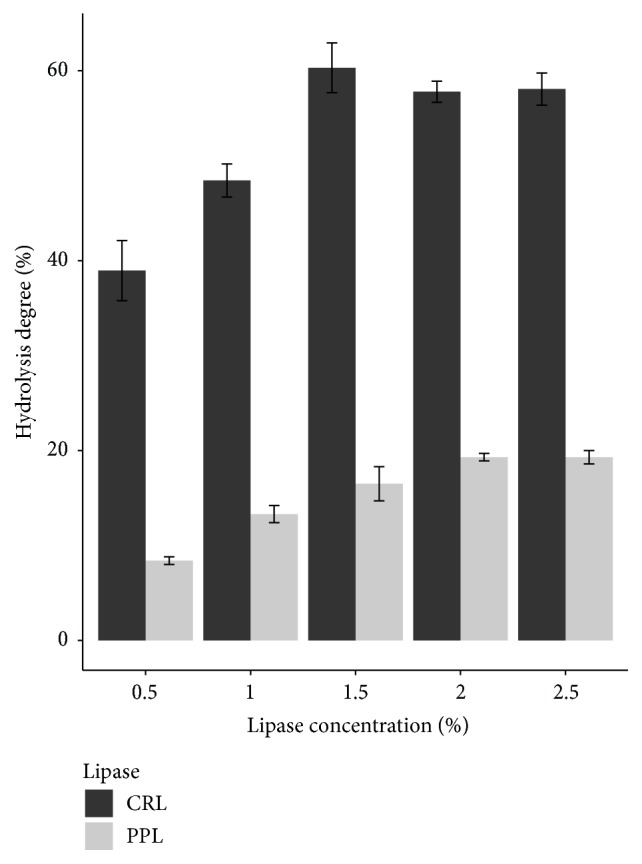
Effect of lipase concentration on hydrolysis degree of two lipase types.

In Figure 2, the highest hydrolysis degree of VCO was achieved at 2% of PPL concentration. Meanwhile, 1.5% of CRL concentration was enough to get the highest hydrolysis percentage. This showed that catalytic ability of CRL was stronger than PPL.

This result also was not equivalent to other researches, similar to the result of oil to buffer ratio, depending on the substrate specificity, and the method of hydrolysis process will lead to the difference in lipase concentration. The study of Sharma et al. (2013) using the substrate as cod liver oil gave the best result at 2% of CRL concentration [7]. And in another research of Freitas et al. (2007), the amount of CRL and PPL to hydrolyze soybean oil was 1% of lipase concentration [11].

### 3.3. Evaluation of pH

Determining suitable pH value for each lipase is necessary because pH has a significant effect on hydrolytic ability of lipase. Dramatic changes in pH (very high or very low) may affect the ionization of the substrate, lipase, or lipase-substrate complex. As a result, the breakdown of substrate and the denaturation of lipase lead to a decrease of hydrolysis degree [13].

**Figure 3:**
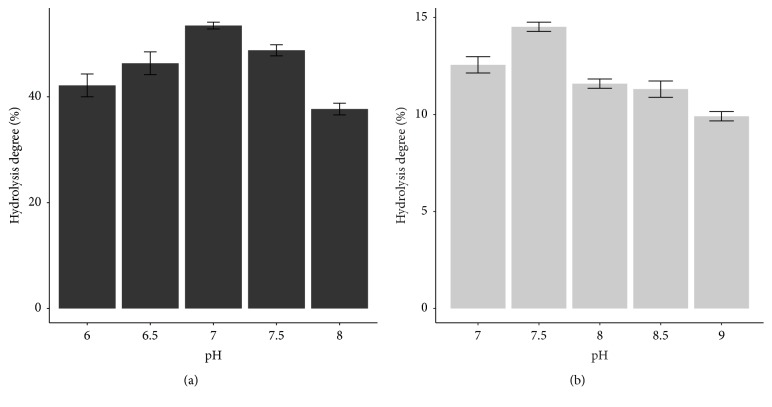
Effect of pH on hydrolysis degree of (a) CRL and (b) PPL.

The highest hydrolysis degree was obtained at pH 7 for CRL (Figure 3(a)) and 7.5 for PPL (Figure 3(b)). Overall, PPL prefers to catalyze hydrolysis reaction in slightly alkaline medium; meanwhile CRL was suitable at neutral pH.

Some other results were equivalent to this study, for example, the hydrolysis of cod liver oil using CRL was conducted at pH 7.0 [7]. Hydrolysis of soybean oil using CRL and PPL at pH 7 and 7.5, respectively, was carried out by Freitas et al. (2007) [11]. Hermansyah et al. (2006) also used CRL to hydrolyze triolein at pH 7.0 [14]. Study of Zou et al. (2010) in the comparison between free PPL and PPL immobilized on ionic liquid modified mesoporous silica SBA-15 showed the optimum pH condition for free PPL at 7.5 [15].

### 3.4. Evaluation of Temperature

Temperature has a strong effect on hydrolysis reaction of lipase. When increasing the temperature of the hydrolysis reaction, viscosity of the mixture is decreased and molecules of lipase become flexible, leading to the advantage of linking lipase to substrate. However, the deactivation of lipase will occur if temperature is too high because the nature of enzyme is protein, leading to a fall of hydrolysis degree.

**Figure 4:**
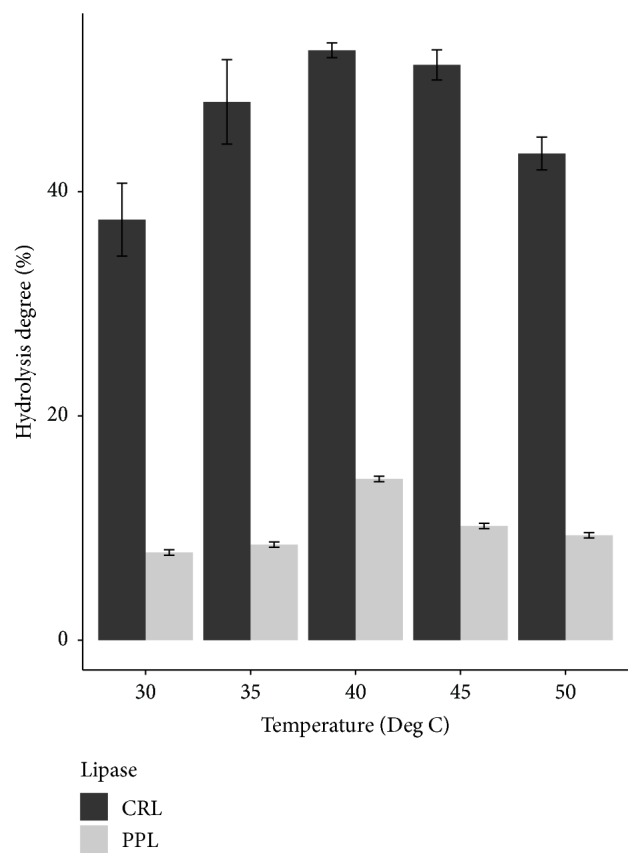
Effect of temperature on hydrolysis degree of two lipase types.

As shown in Figure 4, the hydrolysis degree of both two lipase types achieved the highest rate at temperature of 40°C. This temperature was also equivalent to the study of Freitas et al. (2007) in using CRL and PPL to hydrolyze soybean oil [11]. Zhou et al. (2015) hydrolyzed* Jatropha* oil for biodiesel production using CRL at 40°C [16]. Improving the catalytic hydrolysis reaction of sol-gel-encapsulated CRL was conducted by Ozyilmaz et al. (2014). In the comparison with encapsulated CRL, free CRL was used with the best temperature of 40°C [17]. In the study using PPL to hydrolyze* Mimusops elengi* and* Parkinsonia aculeata* seed oils, Sharma et al. (2009) set up hydrolysis process at 40°C [18].

### 3.5. Hydrolysis Reaction Time

**Figure 5:**
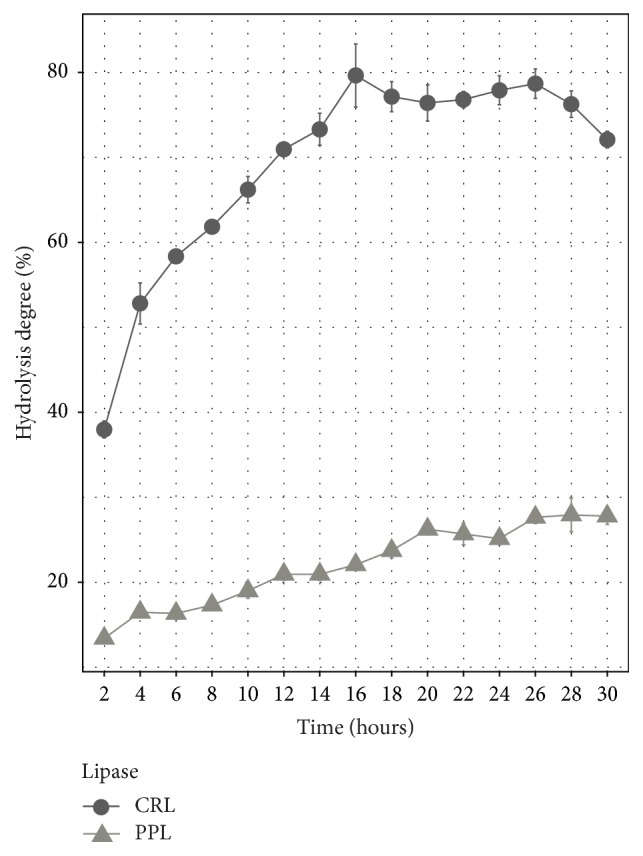
Hydrolysis time of two lipase types.

The hydrolysis degree of two lipase types during 30 hours was shown in Figure 5. Hydrolysis degree of VCO using PPL reached 27.64% after 26 hours of hydrolysis reaction, whereas CRL not only got the higher hydrolysis degree but also took less time than PPL, achieving 79.64% after 16 hours. In this study, the hydrolysis VCO by CRL took less time than other studies. For example, in the study of Freitas et al. (2007) in the hydrolysis process of soybean oil, the highest proportion obtained was 70% for CRL and 23% for PPL [11]. Isooctane as a solvent to dissolve VCO decreases viscosity as well as increasing the interface of VCO and water. Therefore, there are more substrates for lipase working and the hydrolysis time will be shortened, leading to the increase in hydrolysis degree [12].

Overall, CRL gave a higher catalytic activity; not only was the percentage of FFAs released using CRL more than triple compared to PPL but also the hydrolysis time was shorter than PPL. It can be explained that PPL is positional specific lipase; it only hydrolyzes triglycerides in sn-1,3 position and keeps sn-2 position, whereas CRL is a nonspecific lipase which hydrolyzes completely all three ester bonds in triglycerides. Therefore, hydrolysis degree of CRL was more than PPL [18].

### 3.6. Hydrolysis Degree at the First 4 Hours of Hydrolysis Reaction

Overall, there are some differences in hydrolysis degree at different time of reaction. After the first half an hour, the hydrolysis degree of PPL obtained was 7.56%; it saw a slight growth every half an hour, averaging from 1% to 2%, reaching 17.87% after 4 hours of reaction, whereas the hydrolysis degree of CRL was higher than PPL after 0.5 hours, at 11.58%. It continues to increase sharply to more than 2 times after 1.5 hours. Moreover, the hydrolysis degree was doubled at 2 hours, reaching 50.37%. Then it experienced a gradual increase at the other timelines.

**Figure 6:**
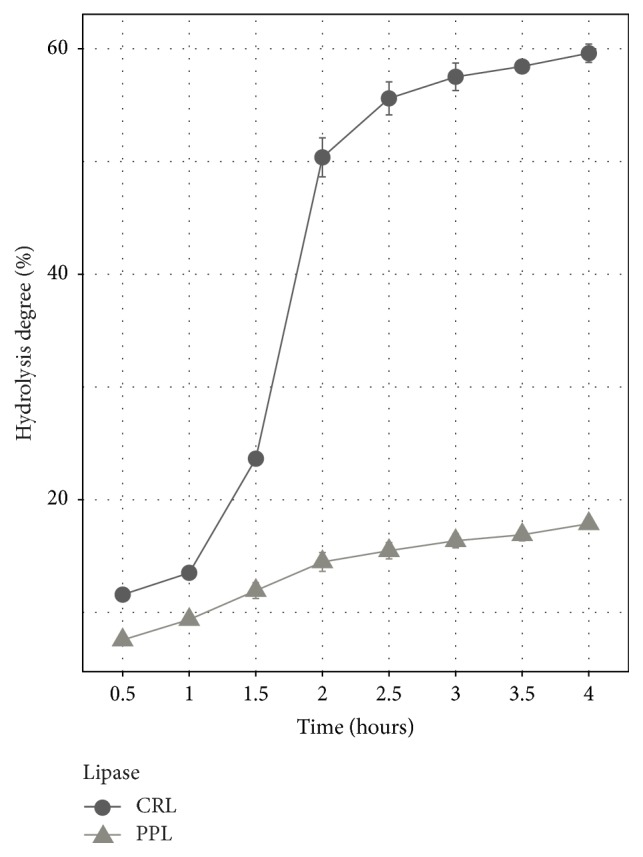
Hydrolysis degree at different time course.

In conclusion, the hydrolysis degree of PPL showed a stable increase but CRL increased significantly from 1.5 to 2 hours. CRL showed higher ability than PPL in catalyst for hydrolysis reaction (Figure 6).

### 3.7. FFAs Composition

**Figure 7:**
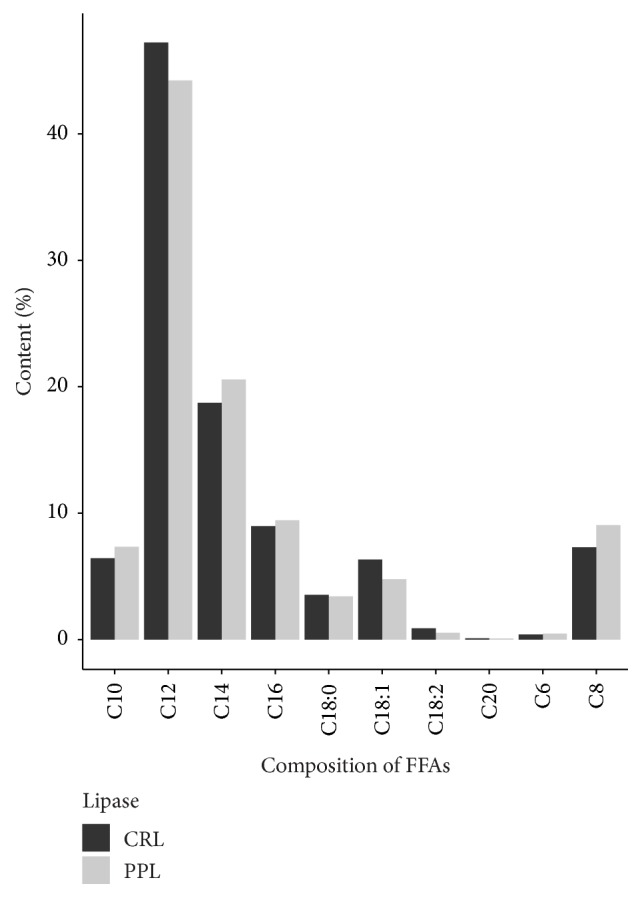
Composition of FFAs obtained after finishing the hydrolysis reaction.

Figure 7 showed that MCFAs were the primary composition of FFAs in which lauric acid (C12) was the largest segment. The hydrolysis reaction using CRL released 47.23% of lauric acid compared to 44.23% for PPL. Although PPL is specific lipase at sn-1,3 position, the percentage of each fatty acid released by PPL was relatively equivalent to CRL (nonspecific lipase).

## 4. Conclusion

The parameters for hydrolysis reaction of VCO using CRL and PPL were determined at VCO to buffer ratios 1 : 5 and 1 : 4, lipase concentrations 1.5% and 2%, pH values 7 and 7.5, and the same temperature at 40°C, respectively. pH condition and temperature were relative to natural properties of each lipase. Moreover, it also was equivalent to previous studies carried out at these pH and temperature. VCO to buffer ratio and lipase concentration showed differences because of substrate specificity of lipase and hydrolysis method. In the comparison of hydrolysis rate of these two lipase types, the hydrolysis degree of CRL increases sharply at the first 2 hours of reaction while PPL showed a slight rise in hydrolysis degree during the first 4 hours of reaction.
